# Risk of End-Stage Renal Disease in Psoriatic Patients: Real-World Data from a Nationwide Population-Based Cohort Study

**DOI:** 10.1038/s41598-019-53017-4

**Published:** 2019-11-12

**Authors:** Eun Lee, Ju Hee Han, Chul Hwan Bang, Seung Ah Yoo, Kyung Do Han, Ha-Na Kim, Young Min Park, Jun Young Lee, Ji Hyun Lee

**Affiliations:** 1 Department of Dermatology, Namcheon Hospital, Gunpo-Si, Korea; 2 Department of Dermatology, Seoul St. Mary’s Hospital, College of Medicine, The Catholic University of Korea, Seoul, Korea; 3 Department of Biostatistics, College of Medicine, The Catholic University of Korea, Seoul, Korea; 4 Department of Family Medicine, St. Vincent’s Hospital, college of Medicine, The Catholic University of Korea, Seoul, Korea

**Keywords:** Diseases, Psoriasis

## Abstract

Psoriasis is a chronic inflammatory skin disorder mediated by the T-cell–related immune response. Psoriatic patients may have a variety of comorbidities, but their risk of end-stage renal disease (ESRD), particularly according to the subtype of psoriasis, is unclear. We investigated the risk of ESRD in patients with psoriasis according to the subtype of psoriasis and history of systemic therapy for psoriasis. A total of 2,121,228 adults (1,590,921 in the control group and 530,307 in the psoriasis group) were enrolled in this nationwide population-based cohort study until 2015. During follow-up, 1,434 of the subjects in the psoriasis group developed ESRD. After adjusting for confounding factors, psoriasis was associated with the risk of ESRD (hazard ratio (HR) 1.58, 95% confidence interval [95% CI] 1.47–1.68). The psoriatic arthritis group (HR 7.60, 95% CI 1.90–30.41) had a higher risk of ESRD than the control group. Interestingly, no such association was detected in the systemically treated group (HR 1.07, 95% CI 0.80–1.41). Moreover, the acitretin-treated group had a lower risk of ESRD (HR 0.658, 95% CI, 0.494–0.875) than the non-systemically treated group. In conclusion, the risk of developing ESRD in patients with psoriasis differed according to the type of treatment and the presence of arthritis.

## Introduction

Psoriasis is a chronic inflammatory skin disease related to T-cell–mediated immunity^1^. The global prevalence of psoriasis is 0.91–8.5%, but it is lower in Asia, including in the Republic of Korea (ROK)^2–7^. Despite novel treatments, psoriasis is associated with a considerable socioeconomic burden and with a variety of comorbidities, which reduce the quality of life of psoriatic patients^8^.

Psoriasis is a serious and refractory disease due to “invisible inflammation”. Psoriasis is not limited to the skin; the “psoriatic march” leads to elevated levels of oxidative stress, endothelial dysfunction, abnormal glucose metabolism, insulin resistance, and ultimately cardiovascular disease^9–11^. Several studies have evaluated the relationship between psoriasis and metabolic, kidney or cardiovascular disease^10,12–14^, but none have addressed the relationships between the subtype of, or treatment for, psoriasis and chronic kidney diseases such as end-stage renal disease (ESRD).

In a cross-sectional study, Yang *et al*. demonstrated a link between psoriasis and chronic kidney disease^15^. Wang *et al*. showed that patients with ESRD on chronic hemodialysis are at increased risk of psoriasis^16^. Moreover, psoriatic patients are at risk of renal disease, the magnitude of which increases with worsening severity of psoriasis^17–19^. Renal dysfunction caused by systemic treatment for psoriasis as well as by psoriasis itself is a concern, but few large-scale studies have investigated the risk of ESRD according to systemic treatment for psoriasis. Therefore, we investigated the actual relationship between details of psoriasis and the prospective risk of ESRD using the National Health Insurance Service (NHIS) database.

## Materials and Methods

### Data source

All citizens of the ROK are enrolled in the NHIS. The NHIS database contains diagnostic codes, prescriptions, patient information, and data on billing for medical expenses^7^. All citizens of the ROK are registered using the standard International Classification of Diseases (ICD)-10 code and receive a unique identification for anonymity number. This study was approved by the Research Committee of the Korean National Health Insurance Service (KNHIS-2018-1-225), and its design, which is in compliance with the principles of the Declaration of Helsinki, was approved by the Ethics Committee of Seoul St. Mary’s Hospital, the Catholic University of Korea (KC18ZESI 0415). The informed consent was waived because all data were anonymized and de-identified.

### Study population

The psoriasis group comprised all patients diagnosed with psoriasis or psoriatic arthritis (ICD-10 codes L40 and M073, respectively) from January 2010 to December 2013 (n = 2,121,228). We excluded patients younger than 20 years of age, those diagnosed with psoriasis before enrollment, and patients who had ESRD before being diagnosed with psoriasis. Finally, the psoriasis group consisted of 530,307 subjects. The control group comprised 1,590,921 subjects (three per patient with psoriasis) selected at random and age- and gender-matched with the subjects in the psoriasis group. We also divided the subjects into the non-systemically treated group (subjects who did not receive a systemic agent) and systemically treated group (subjects who received a systemic agent such as acitretin, cyclosporin, methotrexate, or a biologic [adalimumab, etanercept, infliximab, or ustekinumab])^7^. During the follow-up period (up to 2015), the primary endpoint was newly diagnosed ESRD as defined by the presence of at least one claim per year under an ICD-10 code of N18, N19, Z49, Z94.0, or Z99.2, and a procedure code of R3280 (kidney transplantation), O7011–7020 (hemodialysis), V001 (hemodialysis), O7071-7075 (peritoneal dialysis), or V003 (peritoneal dialysis).

### Statistical analysis

The demographic characteristics of the subjects at baseline are presented as means ± standard deviations or as numbers (%). The incidence of ESRD was calculated as the number of incident cases during the follow-up period (per 1,000 person-years). The incidence of ESRD according to psoriasis, systemic therapy for psoriasis, and psoriatic arthritis was calculated using the Kaplan–Meier method. Differences between groups were analyzed using the log-rank test. The association between subgroups and ESRD was assessed using univariate and multivariate Cox proportional hazard regression analyses. The latter was used to control for the effects of age, gender, income level, region, diabetes, hypertension, and dyslipidemia. Statistical analyses were performed using SAS software (ver. 9.4, SAS Institute, Cary, NC, USA). *P*-values < 0.05 were regarded as indicative of statistical significance.

## Results

Using the NHIS database, we identified 1,590,921 controls (control group) and 530,307 patients with psoriasis (psoriasis group) (Table 1). The baseline characteristics of the groups are shown in Table 1. The prevalence of diabetes, hypertension, and dyslipidemia differed significantly between the two groups (*P* < 0.01).

**Table 1:** Characteristics of the study population at baseline.

Parameter	Psoriasis		*P*
	No	Yes	
	*n* = 1,590,921	*n* = 530,307	
Age, years	42.84 ± 20.21	42.84 ± 20.21	1
**Gender, n (%)**			**1**
Male	817,599 (51.39)	272,533 (51.39)	
Female	773,322 (48.61)	257,774 (48.61)	
**Region, n (%)**			**<0.0001**
Urban	727,878 (45.75)	236,052 (44.51)	
Rural	863,043 (54.25)	294,255 (55.49)	
**Income (low 20%), n (%)**		**<0.0001**	
No	1,150,829 (72.34)	378,368 (71.35)	
Yes	440,092 (27.66)	151,939 (28.65)	
**Diabetes mellitus, n (%)**		**<0.0001**	
No	1,488,188 (93.54)	487,203 (91.87)	
Yes	102,733 (6.46)	43,104 (8.13)	
**Hypertension, n (%)**			**<0.0001**
No	1,321,373 (83.06)	427,832 (80.68)	
Yes	269,548 (16.94)	102,475 (19.32)	
**Dyslipidemia, n (%)**			**<0.0001**
No	1,436,920 (90.32)	464,010 (87.5)	
Yes	154,001 (9.68)	66,297 (12.5)	

The psoriasis group had a high risk of developing ESRD (hazard ratio [HR] 1.81, 95% confidence interval [95% CI] 1.69–1.93, *P* < 0.001), and this association remained significant after adjustment for age, gender, household income, region, and comorbidities of diabetes, hypertension, and dyslipidemia (HR 1.58, 95% CI 1.47–1.68, *P* < 0.001) (Table 2).

**Table 2:** Unadjusted and adjusted HR and 95% CI of ESRD according to psoriasis.

Psoriasis	*n*	Events	Duration	Incidence (per 1,000)	Unadjusted	Model 1	Model 2
No	1,590,921	2,395	6,266,591.16	0.38	1 (Ref.)	1 (Ref.)	1 (Ref.)
Yes	530,307	1,434	2,075,847.75	0.69	1.81 (1.69–1.93)	1.82 (1.71–1.95)	1.58 (1.47–1.68)

Model 1 is adjusted for age and gender.

Model 2 is adjusted for age, gender, household income, region, diabetes mellitus, hypertension, and dyslipidemia.

**Figure 1:**
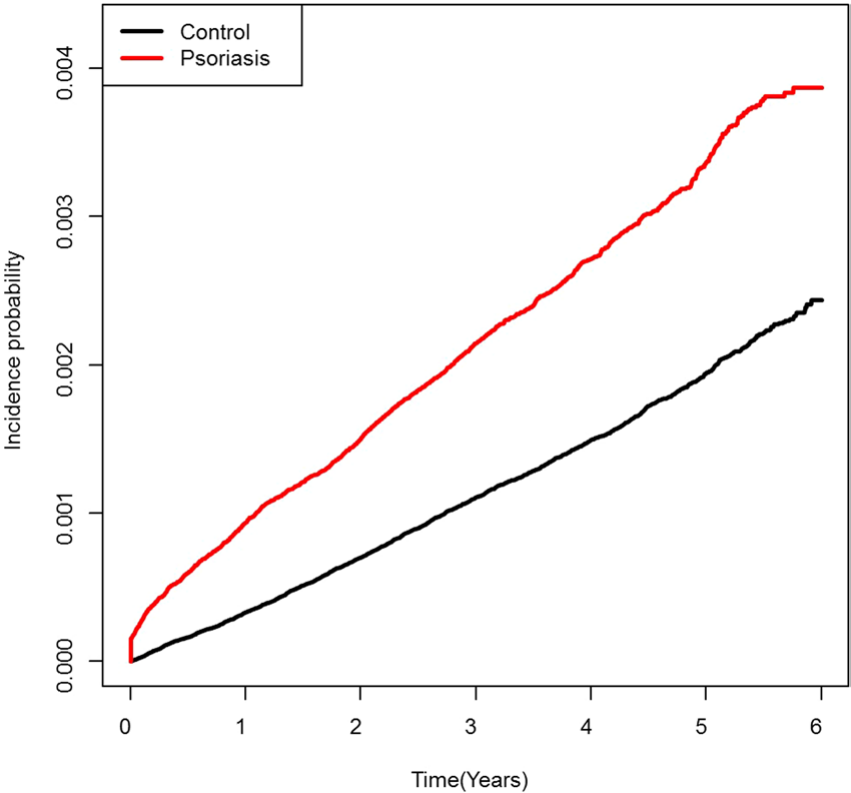
Kaplan–Meier plot of overall end-stage renal disease (ESRD)-free survival in the psoriasis and control groups. The log-rank *P*-value indicates a significant difference in the incidence of ESRD between the two groups.

Kaplan–Meier curves of the ESRD-free survival rate are shown in Fig. 1. The median follow-up duration was 3.9 years. The incidence of ESRD was 0.69 and 0.38 per 1,000 person-years in the psoriasis and control groups, respectively (*P* < 0.001 by log-rank test; Fig. 1).

The results of analyses according to the subtype of psoriasis are listed in Table 3. In the cutaneous psoriasis group, 499,593 patients had not been treated with a systemic agent (non-systemically treated group), 30,535 had been treated with a systemic agent (systemically treated group), and 179 patients were diagnosed with psoriatic arthritis. The non-systemically treated group (HR 1.85, 95% CI 1.73–1.97, *P* < 0.001) and psoriatic arthritis group (HR 7.50, 95% CI 1.89–29.70, *P* < 0.001) had increased risks of ESRD compared to the control group. After adjusting for confounding factors, the significant association with the risk of ESRD remained in the non-systemically treated group (HR 1.60, 95% CI 1.50–1.71, *P* < 0.001) and psoriatic arthritis group (HR 7.60, 95% CI 1.90–30.41, *P* < 0.001).

**Table 3:** Unadjusted and adjusted HR and 95% CI of ESRD according to psoriasis subtype.

Subtype	*N*	Events	Duration	Incidence (per 1,000)	Unadjusted	Model 1	Model 2
Psoriasis, No	1,590,921	2,395	6,266,591.16	0.38	1 (Ref.)	1 (Ref.)	1 (Ref.)
**Psoriasis, Yes**							
**Cutaneous psoriasis**							
Non-systemically treated group	499,593	1,383	1,958,118.73	0.71	1.85 (1.73–1.97)	1.85 (1.74–1.98)	1.6 (1.5–1.71)
Systemically treated group	30,535	49	117,018.43	0.42	1.1 (0.83–1.46)	1.23 (0.93–1.64)	1.07 (0.8–1.41)
Psoriatic arthritis	179	2	710.59	2.81	7.5 (1.89–29.7)	8.06 (2.02–32.08)	7.6 (1.9–30.41)

Non-systemically treated group, subjects who did not receive a systemic agent.

Systemically treated group, subjects treated with a systemic agent (including biologics).

Model 1 is adjusted for age and gender.

Model 2 is adjusted for age, gender, household income, region, diabetes mellitus, hypertension, and dyslipidemia.

Of the subjects, 15,212 had been treated with acitretin (acitretin group) (HR 0.576, 95% CI 0.385–0.863); these subjects did not have a higher risk of ESRD than those in the non-systemically treated group (Table 4). Finally, treatment with methotrexate (HR 0.735, 95% CI 0.329–1.638) or cyclosporin (HR 0.717, 95% CI 0.47–1.092) was not associated with the risk of ESRD.

**Table 4:** Unadjusted and adjusted HR and 95% CI for ESRD according to systemic treatment.

Group	*n*	Events	Duration	Incidence	Unadjusted	Model 1	Model 2
**Cutaneous psoriasis**							
Non-systemically treated group	499,593	1,383	1,958,118.73	0.70629	1 (Ref.)	1 (Ref.)	1 (Ref.)
Systemically treated group	30,535	49	117,018.43	0.41874	0.592 (0.445–0.787)	0.657 (0.494–0.874)	0.658 (0.494–0.875)
Acitretin	15,212	24	61,074.67	0.39296	0.559 (0.373–0.836)	0.564 (0.377–0.844)	0.576 (0.385–0.863)
Methotrexate	3,384	6	13,351.53	0.44939	0.637 (0.286–1.421)	0.699 (0.313–1.559)	0.735 (0.329–1.638)
Cyclosporine	14,200	22	51,233.09	0.42941	0.603 (0.396–0.919)	0.756 (0.496–1.152)	0.717 (0.47–1.092)
Anti-TNF-α	45	0	194.72	0	—	—	—

Non-systemically treated group, subjects not treated with a systemic agent.

Systemically treated group, subjects treated with a systemic agent (including biologics).

Model 1 is adjusted for age and gender.

Model 2 is adjusted for age, gender, household income, region, diabetes mellitus, hypertension, and dyslipidemia.

## Discussion

In this nationwide cohort study, patients with psoriasis showed an increased risk of ESRD development (HR 1.58, 95% CI 1.48–1.68) after adjustment for confounding factors. However, the patients who received systemic anti-psoriasis agents did not have a significantly increased risk of ESRD. Notably, patients treated with acitretin (acitretin group) (HR 0.576, 95% CI 0.385–0.863) significantly did not have an increased risk of ESRD compared to the non-systemically treated group. Meanwhile, psoriatic arthritis was strongly associated with the risk of ESRD (HR 7.60, 95% CI 1.90–30.41).

Psoriasis is a multi-organ rather than a skin-only inflammatory disease^9^. Patients with psoriasis are at high risk of systemic disorders, such as metabolic syndrome, hypertension, cardiovascular diseases, inflammatory bowel diseases, and cancer^10,20–23^. In addition, associations between psoriasis and kidney disease (*e*.*g*., glomerulonephritis, IgA nephropathy, focal segmental glomerulosclerosis, and membranous nephropathy) have been reported^24–26^. The prevalence of microalbuminuria and the mean urinary albumin excretion (an indicator of subclinical renal impairment) are higher in patients with psoriasis than in healthy controls^27,28^. The risk of chronic kidney disease and ESRD is increased in psoriatic patients, especially including those with severe psoriasis, psoriatic arthritis, and patients taking non‐steroidal anti‐inflammatory drugs^17–19^. In this study, there was strongly associated with the risk of ESRD (HR 7.60, 95% CI 1.90–30.41, *P* < 0.001) in patients with psoriatic arthritis as well as those with psoriasis.

Several pathogenetic mechanisms have been proposed to be shared by psoriasis and ESRD. First, the altered serum levels of various factors in psoriatic patients may induce renal impairment, and tubular injury may be caused by elevated levels of uric acid^29^. Second, comorbidities of psoriasis, such as atherosclerosis, arterial hypertension, and diabetes, may contribute to renal dysfunction. Third, T-cell dysfunction and increased levels of immune complexes typically seen in patients with psoriasis are reportedly related to glomerular injury^30^.

Among the various proinflammatory factors associated with both ESRD and psoriasis, interleukin (IL)-17 is notable. IL-17 levels are high in psoriasis skin lesions and in the serum of psoriatic patients^31–33^. Also, the severity of psoriasis, as indicated by the PASI score, is positively correlated with the serum level of IL-17^34^. IL-17A also plays a role in the development of kidney diseases, including glomerulonephritis, nephrotic syndrome, diabetic nephropathy, and acute renal allograft rejection, as well as in atherosclerosis and hypertension^35^. Patients with ESRD have immune impairment, as indicated by an increased abundance of IL-17A–producing effector memory T cells and decreased abundance of naïve T cells^36^. Indeed, the numbers of Th17 cells and Treg cells were reported to be increased and decreased, respectively, in patients on long-term hemodialysis^31^. Therefore, the sustained high serum levels of IL-17 in psoriatic patients may induce renal inflammation and ultimately ESRD.

Systemic treatments such as acitretin, methotrexate, and etanercept decrease the numbers of proinflammatory cells (such as Th1 and Th17 cells) and increase those of anti-inflammatory cells (such as Tregs) in psoriatic skin lesions and in serum^32,33,37,38^. Acitretin downregulates the expression of interferon-γ as well as that of IL-17^32^. Therefore, the levels of proinflammatory cytokines may have been lower in the subjects in the systemically treated group than those in the non-systemically treated group. Cyclosporin is reportedly nephrotoxic^39^, but in this study, cyclosporin did not impact the risk of ESRD. This may be due to the small number of patients treated with cyclosporin and/or the low doses administered, or to the alert regarding the danger of administering cyclosporin to patients with reduced renal function or hypertension.

The greatest strength of this study lies in the analysis of data from a large longitudinal cohort of subjects. To our knowledge, this is the first nationwide cohort study on the relationship of psoriasis with/without psoriatic arthritis and systemic treatment with the risk of ESRD. However, this study had the following limitations. First, disease diagnosis was assessed using the ICD-10 codes in a health-insurance claims database, and the nature of claims data makes coding errors or misclassification possible. Second, because such databases contain limited clinical information, some confounding factors, such as smoking status, drinking status, body mass index, and physical activity level, could not be analyzed. Third, data on the severity of psoriasis could not be obtained.

In conclusion, the risk of ESRD was increased in patients with psoriasis or psoriatic arthritis but not in those who received systemic treatment for psoriasis. These findings provide insight into the relationship between psoriasis and the risk of ESRD.

## References

[1] Nestle FO, Kaplan DH, Barker J (2009). Psoriasis. N Engl J Med.

[2] Griffiths CEM (2017). The global state of psoriasis disease epidemiology: a workshop report. Br J Dermatol.

[3] Chang YT (2009). Epidemiological study of psoriasis in the national health insurance database in Taiwan. Acta dermato-venereologica.

[4] Oh, E., H., Ro, Y. S. & Kim, J. E. Epidemiology and cardiovascular comorbidities in patients with psoriasis: A Korean nationwide population-based cohort study. *J Dermatol* (2017). 10.1111/1346-8138.13761 28191654

[5] Kubota K (2015). Epidemiology of psoriasis and palmoplantar pustulosis: a nationwide study using the Japanese national claims database. BMJ Open.

[6] Ding X (2012). Prevalence of psoriasis in China: a population-based study in six cities. Eur J Dermatol.

[7] Han JH (2018). Epidemiology and Medication Trends in Patients with Psoriasis: A Nationwide Population-based Cohort Study from Korea. Acta Derm Venereol.

[8] Raaby L, Ahlehoff O, de Thurah A (2017). Psoriasis and cardiovascular events: updating the evidence. Arch Dermatol Res.

[9] Boehncke WH, Boehncke S, Tobin AM, Kirby B (2011). The ‘psoriatic march’: a concept of how severe psoriasis may drive cardiovascular comorbidity. Exp Dermatol.

[10] Kim HN, Han K, Song SW, Lee JH (2018). Hypertension and risk of psoriasis incidence: An 11-year nationwide population-based cohort study. PLoS One.

[11] Rhee TM (2017). Increased Risk of Atrial Fibrillation and Thromboembolism in Patients with Severe Psoriasis: a Nationwide Population-based Study. Sci Rep.

[12] Rodriguez-Zuniga MJM, Garcia-Perdomo HA (2017). Systematic review and meta-analysis of the association between psoriasis and metabolic syndrome. J Am Acad Dermatol.

[13] Neimann AL (2006). Prevalence of cardiovascular risk factors in patients with psoriasis. J Am Acad Dermatol.

[14] Lee JH (2018). Psoriasis risk in patients with diabetic retinopathy: A nationwide population-based study. Sci Rep.

[15] Yang YW, Keller JJ, Lin HC (2011). Medical comorbidity associated with psoriasis in adults: a population-based study. Br J Dermatol.

[16] Wang CC, Tang CH, Huang KC, Huang SY, Sue YM (2018). Increased risk of incident psoriasis in end-stage renal disease patients on chronic hemodialysis: A nationwide population-based cohort study. J Dermatol.

[17] Wan J (2013). Risk of moderate to advanced kidney disease in patients with psoriasis: population based cohort study. BMJ.

[18] Chi CC (2015). Risk of incident chronic kidney disease and end-stage renal disease in patients with psoriasis: A nationwide population-based cohort study. J Dermatol Sci.

[19] Chiu HY (2015). Increased risk of glomerulonephritis and chronic kidney disease in relation to the severity of psoriasis, concomitant medication, and comorbidity: a nationwide population-based cohort study. Br J Dermatol.

[20] Boehncke WH, Schon MP (2015). Psoriasis. Lancet.

[21] Lee, J. H, *et al*. Cancer risk in 892 089 patients with psoriasis in Korea: A nationwide population-based cohort study. *J Dermatol* (2018). 10.1111/1346-8138.14698 30443930

[22] Lee, J. H., Han, K. & Gee, H. Y. The Incidence Rates and Risk Factors of Parkinson’s Disease in Patients with Psoriasis: A Nationwide Population-based Cohort Study. *J Am Acad Dermatol* (2019). 10.1016/j.jaad.2019.07.012 31302182

[23] Bang, C. H., *et al*. Association of Psoriasis With Mental Health Disorders in South Korea. *JAMA Dermatol* (2019). 10.1001/jamadermatol.2019.0315 PMC6506891 31066862

[24] Dervisoglu E, Akturk AS, Yildiz K, Kiran R, Yilmaz A (2012). The spectrum of renal abnormalities in patients with psoriasis. Int Urol Nephrol.

[25] Zadrazil J (2006). IgA nephropathy associated with psoriasis vulgaris: a contribution to the entity of ‘psoriatic nephropathy’. J Nephrol.

[26] Kaji T (1994). Membranous nephropathy associated with psoriasis vulgaris. Clin Nephrol.

[27] Szepietowski JC, Bielicka E, Wasik F, Kopec W, Szepietowski T (2000). Microalbuminuria as a subclinical marker of renal impairment in subjects with psoriasis vulgaris. J Eur Acad Dermatol Venereol.

[28] Cecchi R, Seghieri G, Gironi A, Tuci F, Giomi A (1992). Relation between urinary albumin excretion and skin involvement in patients with psoriasis. Dermatology.

[29] Bruce IN, Schentag CT, Gladman DD (2000). Hyperuricemia in psoriatic arthritis: prevalence and associated features. J Clin Rheumatol.

[30] Kim M, Ko Y, Yeo UC, Kim Y, Oh H (1998). Psoriasis and glomerulonephritis. Clin Exp Dermatol.

[31] Lang CL (2014). Correlation of interleukin-17-producing effector memory T cells and CD4+CD25+Foxp3 regulatory T cells with the phosphate levels in chronic hemodialysis patients. ScientificWorldJournal.

[32] Carretero G (2013). Guidelines for the use of acitretin in psoriasis. Psoriasis Group of the Spanish Academy of Dermatology and Venereology. Actas Dermosifiliogr.

[33] Quaglino P (2011). Th1, Th2, Th17 and regulatory T cell pattern in psoriatic patients: modulation of cytokines and gene targets induced by etanercept treatment and correlation with clinical response. Dermatology.

[34] Yilmaz SB, Cicek N, Coskun M, Yegin O, Alpsoy E (2012). Serum and tissue levels of IL-17 in different clinical subtypes of psoriasis. Arch Dermatol Res.

[35] Cortvrindt C, Speeckaert R, Moerman A, Delanghe JR, Speeckaert MM (2017). The role of interleukin-17A in the pathogenesis of kidney diseases. Pathology.

[36] Chung BH (2012). Increased interleukin-17 producing effector memory T cells in the end-stage renal disease patients. Immunol Lett.

[37] Furuhashi T (2013). Photo(chemo)therapy reduces circulating Th17 cells and restores circulating regulatory T cells in psoriasis. PLoS One.

[38] Priyadarssini, M., Chandrashekar, L. & Rajappa, M. Effect of methotrexate monotherapy on T-cell subsets in the peripheral circulation in psoriasis. *Clin Exp Dermatol* (2018). 10.1111/ced.13795 30294828

[39] Powles AV (1998). Renal function after 10 years’ treatment with cyclosporin for psoriasis. Br J Dermatol.

