# Author Correction: Risk of End-Stage Renal Disease in Psoriatic Patients: Real-World Data from a Nationwide Population-Based Cohort Study

**DOI:** 10.1038/s41598-020-59024-0

**Published:** 2020-02-05

**Authors:** Eun Lee, Ju Hee Han, Chul Hwan Bang, Seung Ah Yoo, Kyung Do Han, Ha-Na Kim, Young Min Park, Jun Young Lee, Ji Hyun Lee

**Affiliations:** 1 Department of Dermatology, Namcheon Hospital, Gunpo-Si, Korea; 2 Department of Dermatology, Seoul St. Mary’s Hospital, College of Medicine, The Catholic University of Korea, Seoul, Korea; 3 Department of Biostatistics, College of Medicine, The Catholic University of Korea, Seoul, Korea; 4 Department of Family Medicine, St. Vincent’s Hospital, college of Medicine, The Catholic University of Korea, Seoul, Korea

Correction to: *Scientific Reports* 10.1038/s41598-019-53017-4, published online 12 November 2019

In the original version of this Article, Young Min Park, Jun Young Lee and Ji Hyun Lee were incorrectly affiliated with ‘Department of Dermatology, Namcheon Hospital, Gunpo-Si, Korea’. The correct affiliation is given below.

Department of Dermatology, Seoul St. Mary’s Hospital, College of Medicine, The Catholic University of Korea, Seoul, Korea.

This error has now been corrected in the PDF and HTML versions of the Article.

